# Type I interferons regulate nitric oxide production in *Brucella abortus*-activated microglia contributing to neuronal death

**DOI:** 10.3389/fimmu.2025.1661395

**Published:** 2025-08-27

**Authors:** Julia Rodríguez, Alex D. Guano, Ignacio Mazzitelli, Vida A. Dennis, Ana M. Rodríguez, Guillermo H. Giambartolomei

**Affiliations:** ^1^ Instituto de Inmunología, Genética y Metabolismo (INIGEM), Consejo Nacional de Investigaciones Científicas y Técnicas (CONICET), Facultad de Farmacia y Bioquímica, Universidad de Buenos Aires, Buenos Aires, Argentina; ^2^ Instituto de Investigaciones Biomédicas en Retrovirus y SIDA (INBIRS), Consejo Nacional de Investigaciones Científicas y Técnicas (CONICET), Facultad de Medicina, Universidad de Buenos Aires, Buenos Aires, Argentina; ^3^ Center for NanoBiotechnology Research and Department of Biological Sciences, Alabama State University, Montgomery, AL, United States

**Keywords:** *Brucella abortus*, neurobrucellosis, microglia, type I interferons, nitric oxide, neuronal death

## Abstract

Microglia have a central role in the immunopathogenesis of neurobrucellosis where its activation is a hallmark in this disease. In this study, we present *in vitro* evidence that type I interferons (IFN) are involved in the activation of microglia during *Brucella abortus* infection and are necessary to induce neuronal death. Neutralization of type I IFN receptor (IFNAR) on microglia cells completely abrogates neuronal loss in primary co-cultures of neurons/microglia infected with *B. abortus* or treated with culture supernatants from *B. abortus*-infected astrocytes. Type I IFN regulate inducible nitric oxide synthase (iNOS) expression, and subsequently nitric oxide (NO) production in microglia by increasing STAT1 expression and phosphorylation. Our results also show that NF-κB and the MAPK signaling pathways, ERK1/2 and p38, are implicated in the secretion of type I IFN induced by the bacterium. Finally, our results indicate that iNOS induction and NO production require activation of both NF-κB and STAT1 transcription factors. This observed molecular mechanism contributes to neuronal death induced by *B. abortus*-activated microglia and may help explain the neurological signs observed in patients with neurobrucellosis.

## Introduction

Despite being a disease with diverse clinical manifestations, inflammation is a hallmark of human brucellosis. This infection, produced by intracellular bacteria *Brucella* spp., progresses causing focalizations in a variety of organ systems. The most morbid form of the disease is known as neurobrucellosis and affects the central and peripheral nervous system. This focalization also presents with inflammatory signs and symptoms (1–3). The importance of this inflammatory disorder is due to the fact that it can lead to irreversible damage in the central nervous system (CNS), with neuronal loss (4); which may be associated with the numerous neurological sequelae described in neurobrucellosis (3–6).

As neuronal dysfunction is further analyzed in light of this evidence, it is important to consider which cells generate an inflammatory milieu that could damage neurons during the invasion of the CNS by *Brucella*. In this respect, we have proved that *B. abortus*-activated microglia induce neuronal demise through primary phagocytosis (also known as phagoptosis) (7, 8). This peculiar mechanism of neuronal death induced by *Brucella*-activated microglia depends on two concomitant events: the release of sublethal amounts of nitric oxide (NO) together with the increased phagocytic capacity of microglia. *B. abortus*-activated microglia secrete NO, which induces neuronal exposure of the “eat-me” signal phosphatidylserine (PS). This signal activates the microglial phagocytosis of alive neurons through the vitronectin receptor, using MFG-E8 as a bridging molecule. Blocking either of the two mechanisms eradicates neuronal death (7, 8).

Microglia, which are the resident macrophages of the CNS, are an exquisitely responsive and highly plastic cell population. They sense and react to the local production of many different signals, including diverse cytokines. In recent years, the impact of microglial cytokine signaling in health and disease has begun to be elucidated. During homeostasis most cytokines are produced at low levels, yet, any insult such as injury, infection or autoimmunity triggers their production. This increment usually contributes to the clearance of the pathogen and/or limit injury, but can in the process exacerbate disease progression. Particularly, microglia are strong responders to interleukin-6 (IL-6) and type I interferons (IFN) (9); two cytokines that are critical in maintaining homeostasis and regulating immune responses. However, their sustained elevated production identifies them as key mediators in the pathogenesis of many neuroinflammatory disorders (9). We have recently untangled the role of IL-6 in the neuronal demise inflicted by *B. abortus*-activated microglia. Our results demonstrated that autocrine microglia-derived and paracrine astrocyte-secreted IL-6 via trans-signaling provides microglial cells with upregulated phagocytic capacity that allows them to kill neurons by phagoptosis (8). However, inhibition of IL-6 signaling in *B. abortus*-activated microglia did not affect NO production. Thus, IL-6 contributes to only one of the two requirements necessary to induce primary phagocytosis of neurons, suggesting that other mediators are involved in regulating NO production in microglia. Since type I IFN have been implicated in the regulation of NO secretion in different models of infectious diseases (10), we aim to investigate if they are also relevant in modulating the NO release in *Brucella*-activated microglia and if they have a role in primary phagocytosis.

## Materials and methods

### Animals

BALB/c mice were acquired at the School of Medicine, University of Buenos Aires, Argentina. Mice were housed in positive-pressure cabinets, maintained under specific-pathogen-free conditions and supplied with sterile food and water *ad libitum*. The temperature was kept at 22 ± 2°C and the lighting was on a 12-hour cycle. National Institute of Health (USA) rules and standards were used to perform all animal procedures, and experiments were approved by the Ethics Committee of Care and Use of Laboratory Animals of the School of Medicine, University of Buenos Aires (Protocol #181/2020).

### Primary cell cultures

Mixed glial cell cultures were obtained from postnatal day 1 to 3 mouse forebrain, and cultures of cortical neurons (>95%) were obtained from embryonic day 16 to 18 mouse forebrain following previously described procedures (7, 11). Mixed glial cell cultures were grown for 2–3 weeks, and then microglia were harvested (>95%) by orbital shaking (2 h at 37°C, 180 rpm) and astrocytes were harvested (>95%) by trypsinization. Co-cultures of neurons/microglia were established by addition of 1 x 10^5^ microglial cells on top of neurons cultures (3 x 10^5^ cells/well in 24-well plates), and allowed to adhere overnight before treatment. Microglia were seeded at a density of 1 x 10^5^ cells/well for phagocytosis assay and confocal microscopy or 1.4 x 10^6^ cells/well for Real-time quantitative PCR (RT-qPCR) and Western blot analyses. Astrocytes were seeded at a density of 3.5 x 10^5^/well (24-well plates) to obtain culture supernatants, or at 1.4 x 10^6^ cells/well for RT-qPCR. Supernatants were used for stimulation of microglia and neurons/microglia cultures. All cell cultures were grown at 37°C and 5% CO_2_ atmosphere in Dulbecco’s modified Eagle’s medium (DMEM) with high glucose, supplemented with 10% fetal bovine serum (FBS), 2 mM L-glutamine, 1 mM sodium pyruvate, 100 U/mL penicillin and 100 μg/mL streptomycin (all reagents from Gibco).

### Bacteria

*B. abortus* S2308 was cultivated for a period of 3 to 5 days in tryptic soy agar (TSA, Merck) at a temperature of 37°C. The bacterial inoculum was prepared by suspending the bacteria in a sterile phosphate-buffered saline (PBS) solution. The number of bacteria was estimated by measuring the optical density of the solution at a wavelength of 600 nm using a spectrophotometer (Amersham Biosciences). Experiments performed with viable bacteria were executed at biosafety level 3 facilities located at the INBIRS (School of Medicine, University of Buenos Aires). To prepare HKBA (heat killed *B. abortus*), microorganisms were washed in PBS, heat-killed at 70°C for 20 min, aliquoted and stored at -70°C until used. Absence of *B. abortus* viability after heat-killing was verified by the lack of bacterial growth on TSA plates.

### Bacterial components

*B. abortus* lipidated outer membrane protein of 19 kDa (L-Omp19) and unlipidated (U)-Omp19 were obtained according to protocols previously described by our laboratory (12). Both recombinant proteins contained less than 0.25 endotoxin U/μg of protein as assessed by *Limulus* Amebocyte Lysates (Associates of Cape Cod Inc.), and were used to stimulate cell cultures at a concentration of 500 ng/mL. Cultures were also stimulated with lipopolysaccharide (LPS) from *E. coli* (100 ng/mL; Sigma-Aldrich) and the synthetic lipohexapeptide tripalmitoyl-S-gliceryl-Cys-Ser-Lys4-OH (Pam_3_Cys) (50 ng/mL; Boehringer Mannheim) to serve as controls.

### *In vitro* infections and culture treatment

Microglia, astrocytes and neurons/microglia cultures were infected with *B. abortus* S2308 at a multiplicity of infection (MOI) of 100 in culture medium without antibiotics for 24 or 48 h. Culture supernatants (SN) from infected astrocytes were collected and sterilized using a 0.22 μm filter (JetBiofil) to remove the remaining bacteria, aliquoted and stored at -70°C until used to stimulate cultures. Microglia and neurons/microglia co-cultures were stimulated for 48 h with SN from non-infected or *B. abortus*-infected astrocytes diluted in complete medium (1/2). Untreated wells were used as negative control.

### Gene expression by RT-qPCR

Extraction of microglia and astrocyte’s total RNA was carried out by Quick-RNA MiniPrep Kit (Zymo Research) following the manufacturer’s instructions. cDNA was synthesized from 1 μg of total RNA using the reverse transcriptase Improm-II enzyme (Promega). RT-qPCR was performed with the master mix FastStart Universal SYBR Green Master^®^ (ROX, Roche) in a StepOne Real-Time PCR System^®^ (Applied Biosystems). Primers used were as follows: IFN-α forward: 5’-TCTGATGCAGCAGGTGGG-3’, reverse: 5’-AGGGCTCTCCAGACTTCTGCT-3’, IFN-β forward: 5’-GCACTGGGTGGAATGAGACT-3’, reverse: 5’-AGTGGAGAGCAGTTGAGGACA-3’, iNOS forward: 5’-CAGCTGGGCTGTACAAACCTT-3’, reverse: 5’-CATTGGAAGTGAAGCGTTTCG-3’, β-actin forward: 5’-AACAGTCCGCCTAGAAGCAC-3’, reverse: 5’- CGTTGACATCCGTAAAGACC-3’. The amplification cycle was as follows: initial denaturation for 10 min at 95°C, followed by 40 cycles at 95°C for 15 s, 60°C for 30 s, and 72°C for 60 s. Melting curve analysis was performed on all primer sets (Invitrogen), yielding a single amplification product. The fold change (relative expression) in gene expression was calculated using the relative quantitation method (2^−ΔΔCt^). The relative expression levels were normalized to the expression of β-actin.

### Neutralization of type I IFN signaling

Neutralization experiments were performed using anti-IFNAR1 monoclonal antibody (10 μg/mL; clone MAR1-5A3; BioLegend) or its isotype control (10 μg/mL; BioLegend), which were added 1 h before infection/stimulation of neurons/microglia co-cultures or microglia cultures.

### Quantification of neuronal density in neurons/microglia co-cultures by fluorescence microscopy

After infection or SN treatment, neurons/microglia co-cultures were dye as previously reported (7). Briefly, cells were washed and fixed with 4% paraformaldehyde (PFA). Then, cells were permeabilized (0.125% v/v Triton X-100) (Promega), followed by a blocking step with PBS containing 5% FBS. Neurons were labeled with anti-β-Tubulin III monoclonal antibody (Sigma-Aldrich) followed by Alexa Fluor 546-labeled anti-mouse IgG2a (Life Technologies Inc.). Microglia were labeled with biotinylated *Griffonia simplicifolia* isolectin-B4 (Vector Laboratories) followed by Alexa Fluor 488-labeled streptavidin (BioLegend). To dye nuclear structures, 4’,6-diamidino-2-phenylindole (DAPI, Molecular Probes) was used and nuclear morphology was analyzed to identify healthy *vs*. apoptotic cells. Images were acquired by a Nikon Eclipse Ti-E PFS microscope and analyzed using ImageJ^®^ software. Five microscopic fields per duplicate coverslip were counted (100–150 neurons). Neuronal viability was calculated with respect to untreated/non-infected controls, and expressed as percentage.

### Nitric oxide determination in culture supernatants by Griess reaction

Nitric oxide (NO) levels in culture supernatants were evaluated by measurement of nitrite concentration (stable metabolite of NO) by colorimetric Griess reaction. For this, 50 μL of culture supernatant was incubated with 50 μL of Griess reactive (1% sulfanilamide and 0.1% naphthylethylenediamine dihydrochloride in 2.5% phosphoric acid) for 10 minutes at room temperature. Absorbance at 550 nm was measured in a microplate reader. Nitrite concentration was determined using a standard curve of sodium nitrite (NaNO_2_).

### Phagocytosis assay

Phagocytic capacity of microglia was evaluated using fluorescent carboxyl microbeads where cells were incubated with 0.003% w/v Nile red 5 - 5.9 μm beads (Spherotech) for 2 h at 37°C and 5% CO_2_. Next, microglia were washed five times with ice-cold PBS to arrest bead uptake and fixed with 4% PFA. Both the number of phagocytic microglia and the number of beads uptake per microglia were determined by fluorescence microscopy.

### Cytokine measurement in culture supernatants

Murine IL-6 and TNF-α were measured in culture supernatants by ELISA following the manufacturer’s instructions (BD Pharmingen). Type I IFN levels were evaluated in culture supernatants using the reporter cell line B16-Blue™ IFN-α/β following the manufacturer’s instructions (InvivoGen). Type I IFN concentrations were determined using a recombinant murine IFN-β (R&D Systems) standard curve.

### Protein determination by western blot

Microglial cell extracts were obtained by cell lysis at 4°C with RIPA buffer (10 mM Tris-HCl pH 7,4; 150 mM NaCl; 1% SDS; 1% sodium deoxycholate, 1% Triton X-100; 5 mM EDTA) plus protease and phosphatase inhibitors cocktails (Sigma-Aldrich). Cell lysates were centrifuged at 14,000 rpm for 15 minutes at 4°C and total protein concentration in extracts was determined by bicinchoninic acid assay (Pierce) using bovine serum albumin as a standard. Equal amounts of total protein (25 μg) were loaded onto SDS-PAGE 10% gel and after electrophoretic separation, proteins were transferred to a nitrocellulose membrane (Amersham GE Healthcare) for 45 minutes at 250 mA. Membrane was blocked for 1 h with 0.1% Tween-20 in TBS 1X and incubated later with primary antibodies anti-iNOS (1:1000 dilution; BD Transduction Laboratories), anti-STAT1 phosphorylated (Tyr701) or total (1:1000 dilution; Cell Signaling Technology), anti-ERK1/2 phosphorylated or total (1:1000 dilution; Santa Cruz Biotechnology), anti-p38 phosphorylated or total (1:1000 dilution; Santa Cruz Biotechnology) and anti-β-actin (1:4000 dilution; clone AC-15; Sigma-Aldrich) overnight at 4°C. β-actin, total p38 and total Erk1/2 were used as loading controls. Next, membrane was washed three times with 0.05% Tween-20 in TBS 1X and incubated with secondary antibodies anti-rabbit IgG or anti-mouse IgG conjugate to peroxidase (1:2000 dilution; Santa Cruz Biotechnology) for 1 h at room temperature. Finally, membrane was revealed using SuperSignal West Femto Maximum Sensitivity Substrate (Thermo Scientific) and chemiluminescent signal was detected by a GeneGnome XRQ System^®^ (Syngene).

### Inhibition of NF-κB and MAPK pathways

Microglia cultures were pre-incubated for 1 h at 37°C with specific inhibitors for NF-κB pathway (BAY 11-7082; 20 μM; Calbiochem) and the MAPK signaling pathways: ERK1/2 (PD98059; 50 μM; Calbiochem), JNK1/2 (SP600125; 10 μM; Calbiochem) y p38 (SB203580; 10 μM; Calbiochem) followed by stimulation with HKBA (1 x 10^8^ bacteria/mL) for 24 or 48 h. Cell viability after incubation with the inhibitors was evaluated by trypan blue exclusion, being higher than 95% in all cases. Culture supernatants were collected to measure NO, type I IFN and TNF-α, as mentioned before. Cell extracts were obtained to evaluate selected proteins by Western blot. When indicated, microglia cultures were also treated with mouse recombinant IFN-β (500, 1000 or 2000 pg/mL; R&D Systems) and culture supernatants were collected to measure NO.

### Quantification of p65 nuclear translocation by confocal microscopy

For subcellular localization analysis of p65 subunit of NF-κB, microglia cultures (1 x 10^5^ cells seeded in glass coverslips of 12 mm diameter) were infected with *B. abortus* S2308 for 24 h in presence or absence of anti-IFNAR1 neutralizing antibody or its isotype control. Cells were washed and fixed with 4% PFA, permeabilized with 0.125% v/v Triton X-100 in PBS for 20 min and blocked with 5% FBS in PBS. Subsequently, p65 subunit was labeled with an anti-p65 monoclonal antibody (1:250 dilution; Santa Cruz Biotechnology) followed by an anti-mouse IgG1 conjugated to Alexa Fluor 546 (1:200 dilution; Life Technologies Inc.). Microglial cells were labeled with B4-isolectin of *Griffonia simplicifolia* (1:500 dilution; Vector Laboratories) followed by Alexa Fluor 488-labeled streptavidin (1:200 dilution; BioLegend). For nuclear staining DAPI was used. Images were acquired using an Olympus FV1000^®^ confocal microscope. CellProfiler^®^ was used to identify the nuclei and cytoplasmic regions of microglia cells (object segmentation), to measure the mean fluorescence intensity in each cell region and to calculate the ratio between these two compartments for each cell. Four microscopic fields per duplicates coverslip were analyzed.

### RNA-seq differential expression analysis of public transcriptome datasets

Microglial RNA-seq public datasets PRJEB46781 (n=3 per group) (13) and GSE75246 (n=5 per group) (14) were downloaded from the European Nucleotide Archive (ENA) and Gene Expression Omnibus (GEO) repositories. Transcript quantification was performed with Kallisto, and gene-level counts were summarized using tximport with Ensembl annotations (GRCm38, release 102). Differential expression analysis was conducted separately for each dataset using DESeq2 R package (15), comparing GFAP-IFN transgenic mice vs. WT mice and LPS injected mice vs. vehicle injected mice. Log2 fold change values for *Nos2*, *Tnf* and *Stat1* genes were calculated for each dataset comparing with its respective control group, and visualized in a heatmap using pheatmap R package.

### Statistical analysis

Experiments were conducted using different primary cultures at least three times, with similar results. Normal distribution of the data within each group was assessed by the Shapiro-Wilk test. Two-way ANOVA followed by the *post hoc* Tukey test were performed within experiments of neuronal viability, one-way ANOVA followed by the *post hoc* Tukey test were performed in experiments comparing more than two groups or two-tailed Student’s t test in experiments comparing two groups; using GraphPad Prism 8.0 software. Data are shown as mean ± SEM from a representative experiment of three performed (each experiment conducted in duplicated).

## Results

### *Brucella abortus* infection induces IFN-α/β expression in astrocytes and microglia

**Figure 1:**
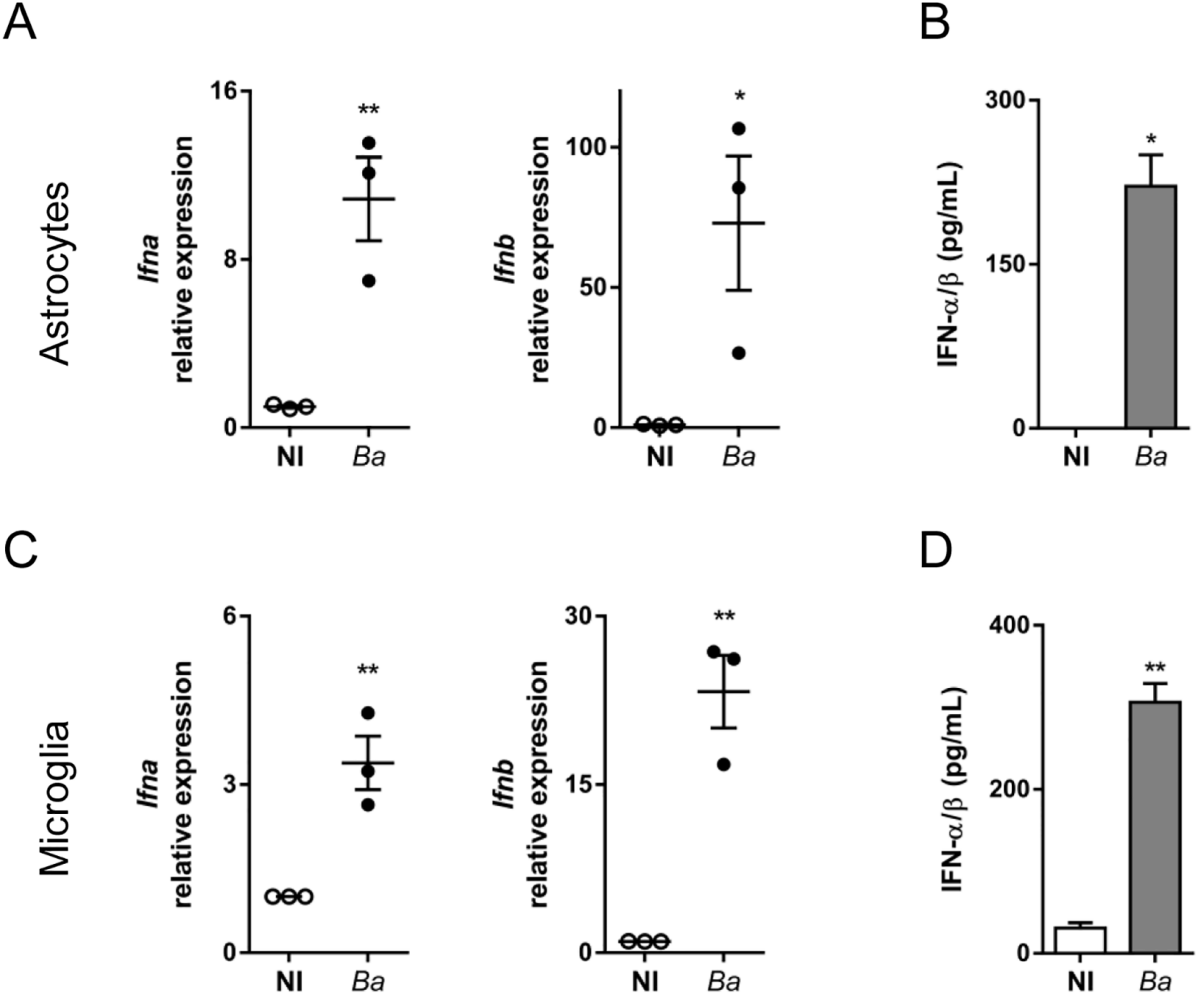
*B abortu*s infection induces IFN-α/β expression in astrocytes and microglia. Cultures of astrocytes **(A, B)** and microglia **(C, D)** were infected with *B. abortus* (*Ba*; MOI 100) for 24 h. Gene expression of *Ifna* and *Ifnb* were determined by RT-qPCR. Each point **(A, C)** represents an independent experiment (mean ± SEM). Secretion of type I IFN was evaluated in culture supernatants using the reporter cell line B16-Blue™ IFN-α/β. Data are shown as mean ± SEM from a representative experiment of three performed **(B, D)**. *p < 0.05; **p < 0.005 *vs*. non-infected (NI) cultures.

To investigate whether *B. abortus* induces type I IFN in glial cells, astrocytes or microglia were infected with the bacteria for 24 h. Gene expression of *Ifna* and *Ifnb* were measured by RT-qPCR, and type I IFN levels in culture supernatants were determined using the reporter cell line B16-Blue™ IFN-α/β. Infection of astrocytes resulted in a significant (*p* < 0.05) increase in both gene expression and secreted type I IFN (**Figures 1A, B**). Likewise, infection of microglia with *B. abortus* also induced a significant (*p* < 0.005) increase of type I IFN genes expression and secretion (**Figures 1C, D**). These findings demonstrate that *B. abortus* induces type I IFN expression in glial cells.

### Neutralization of type I IFN signaling inhibits neuronal death induced by *B. abortus*-activated microglia

**Figure 2:**
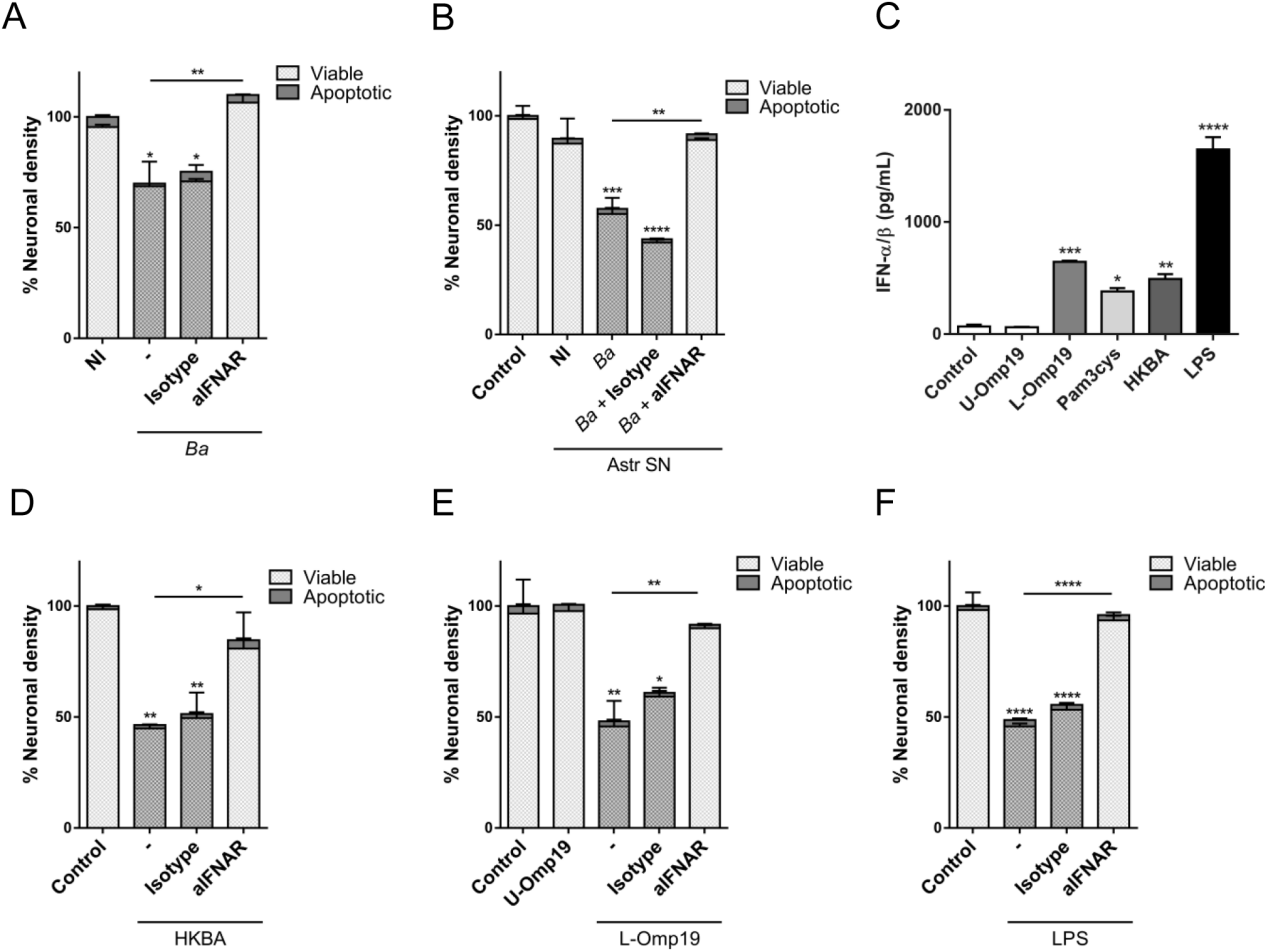
Neutralization of type I IFN signaling inhibits neuronal death induced by *B abortus*-activated microglia. Neurons/microglia co-cultures were infected with *B abortus* (*Ba*; MOI 100) **(A)**, or treated with culture supernatant (1/2 dilution) from non-infected (NI) or *B abortus*-infected *Ba* astrocytes (Astr SN) **(B)** in the presence of an anti-IFNAR1 neutralizing antibody (aIFNAR; 10 μg/mL) or its isotype control (10ug/ml) for 48 h. Co-cultures were fixed and labeled with anti-b-Tubulin III antibody (neurons) and DAPI (nucleus). The percentage (%) of neuronal density was evaluated by fluorescence microscopy and was calculated vs. non-infected (NI) or untreated cultures (Control) **(A, B)**. Microglia cultures were treated with unlipidated outer membrane protein 19 (U-Omp19; 500 ng/mL) or lipidated Omp19 (L-Omp19; 500 ng/mL) from *Ba*, or with either Pam_3_Cys (50 ng/mL), heat-killed *Ba* (HKBA; 1 x 10^8^ bacteria/mL), or LPS from *E coli* (100 ng/mL). Secretion of type I IFN was evaluated in culture supernatants using the reporter cell line B16-Blue™ IFN-α/β **(C)**. Neurons/microglia co-cultures were treated with HKBA (1 x 10^8^ bacteria/mL) **(D)**, U-Omp19 (500 ng/mL) or L-Omp19 (500 ng/mL) from *Ba* **(E)**, and with LPS from *E. coli* (100 ng/mL) **(F)** in the presence of an anti-IFNAR1 neutralizing antibody (aIFNAR; 10 μg/mL) or its isotype control (10 μg/mL) for 48 (h) Neuronal density was evaluated by immunofluorescence. The percentage (%) of viable and apoptotic neurons was calculated as in A and B above. Data are shown as mean ± SEM from a representative experiment of three performed. *p < 0.05; **p < 0.005; ***p < 0.0005; ****p < 0.0001 *vs*. NI or Control cultures, except were indicated.

We have previously shown that *B. abortus* infection or the bystander activation of microglia by culture supernatants from *B. abortus*-infected astrocytes elicits neuronal death by primary phagocytosis (7, 8). To determine whether type I IFN signaling was actually involved in *B. abortus*-induced neuronal loss we pre-incubated neurons/microglia co-cultures with anti-interferon-alpha/beta receptor (IFNAR)1 neutralizing antibodies followed by either infection of microglia with *B. abortus* or their activation by treatment with culture supernatants from *B. abortus*-infected astrocytes. Inhibition of type I IFN signaling resulted in complete abrogation of neuronal death induced by activated microglia. Isotype control antibody had no effect on primary phagocytosis of neurons induced by activated microglia (**Figures 2A, B**).

Our previous results showed that activation of microglia with heat-killed *B. abortus* (HKBA) or the prototypical *B. abortus* lipoprotein Omp19 (L-Omp19), which is recognized through TLR2, are also capable of inducing neuronal death by phagoptosis, increasing both NO production and phagocytic activity in microglia (7). Although, we have previously demonstrated their capacity to induce several pro-inflammatory cytokines in microglia (11), it has not been reported if they are able to induce type I IFN production in these cells. To evaluate this, we determined the production of type I IFN in microglia treated with HKBA or L-Omp19. In parallel, we treated microglia cultures with *E. coli* LPS (TLR4 agonist) as a control given its widely-described ability to induce type I IFN (16). Both, L-Omp19 and HKBA were able to induce type I IFN production in microglia (**Figure 2C**). U-Omp19, the unlipidated protein, was unable to induce type I IFN secretion, indicating that acylation of the protein is necessary in this phenomenon. This was corroborated by stimulation with the lipohexapeptide Pam_3_Cys (TLR2 synthetic agonist), which mimics the lipid portion of bacterial lipoproteins and was also capable of inducing type I IFN production in microglia (**Figure 2C**). Finally, we tested whether neuronal death induced by HKBA or L-Omp19 in neurons/microglia co-cultures is also modulated by type I IFN. Neutralization of IFNAR completely abolished neuronal death caused by activation of microglia with HKBA or L-Omp19 (**Figures 2D, E**), both of them *bona fide* surrogates of infection (7). Moreover, this result was also extended to microglia activated by *E. coli* LPS (**Figure 2F**), which was reported to induce phagoptosis of neurons (17). Overall, these results indicate that type I IFN are indispensable for neuronal phagoptosis to occur.

### IFNAR blocking inhibits NO release of activated microglia, but not its phagocytic capacity

**Figure 3:**
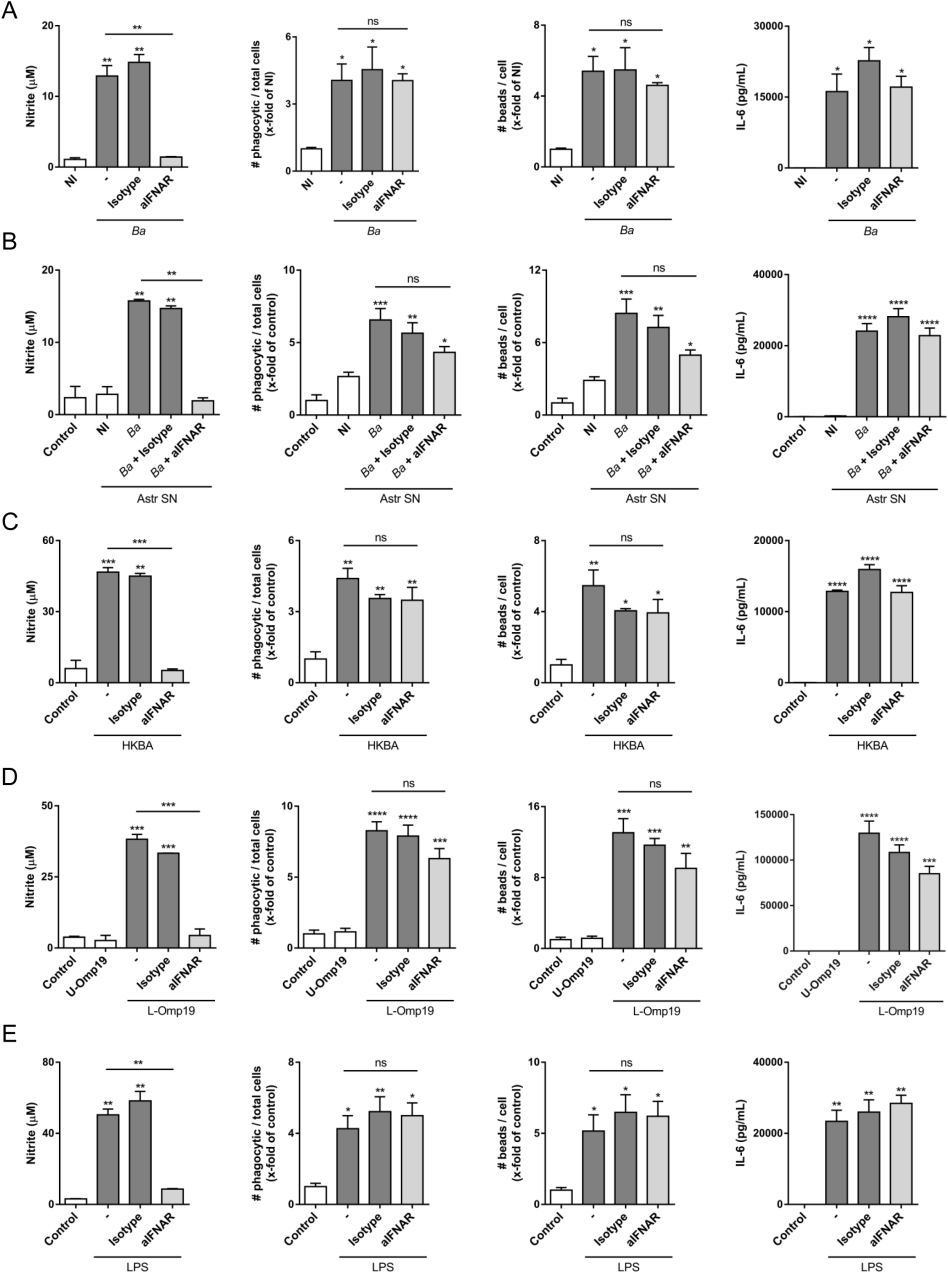
IFNAR neutralization inhibits nitric oxide (NO) release of activated microglia, but not its phagocytic capacity. Microglia cultures were infected with *B abortus* (*Ba*; MOI 100) **(A)**, or treated with culture supernatant (1/2 dilution) from non-infected (NI) or *Ba*-infected (*Ba*) astrocytes (Astr SN) **(B)**, with heat-killed *Ba* (HKBA; 1 x 10^8^ bacteria/mL) **(C)**, unlipidated outer membrane protein 19 (U-Omp19; 500 ng/mL) or lipidated Omp19 (L-Omp19; 500 ng/mL) from *Ba* **(D)**, and with LPS from *E coli* (100 ng/mL) **(E)** in the presence of an IFNAR1 neutralizing antibody (aIFNAR; 10 μg/mL) or its isotype control (10 μg/mL) for 48 h NO release was evaluated in culture supernatants by Griess reaction. Microglial phagocytic activity was determined through a phagocytosis assay with fluorescent microbeads, and visualized by Gene expression of Nos2 was analyzed by RT-qPCR. Fluorescence microscopy. We determined both the number of phagocytic microglia and the number of beads taken per microglia. IL-6 secretion was measured in culture supernatant by ELISA. Data are shown as mean ± SEM from a representative experiment of three performed. *p < 0.05; **p < 0.005; ***p < 0.0005; ****p < 0.0001 *vs*. non-infected (NI) or untreated cultures (Control), except were indicated. Non-significant (ns).

Neuronal death by primary phagocytosis relies on two concomitant events: NO secretion (which triggers the exposure of the “eat-me” signal PS on neurons) and the increased phagocytic capacity of microglia (7, 8). To investigate if type I IFN is involved in regulating NO production in *B. abortus*-activated microglia, we evaluated the generation of this intermediate by using the Griess reaction in microglia cultures infected with *B. abortus* or treated with culture supernatants from *B. abortus*-infected astrocytes, HKBA, L-Omp19 or LPS in the presence of an anti-IFNAR1 neutralizing antibody. Blocking type I IFN signaling resulted in a complete abrogation of NO release in all the evaluated conditions. On the contrary, IFNAR neutralization did not modified the phagocytic capacity of microglia (evaluated as both the number of phagocytic microglia and the uptake of beads per microglia), nor IL-6 secretion (**Figures 3A–E**) which, as we have already demonstrated, is a key cytokine in modulating the phagocytic capacity of microglia (8). Isotype control antibody had no effect on any of the observed phenomena. These results indicate that type I IFN controls NO release from *B. abortus*-activated microglia, but it does not modify its phagocytic activity.

### Neutralizing type I IFN signaling in *B. abortus*-activated microglia suppresses iNOS expression through inhibition of STAT1 expression and phosphorylation

**Figure 4:**
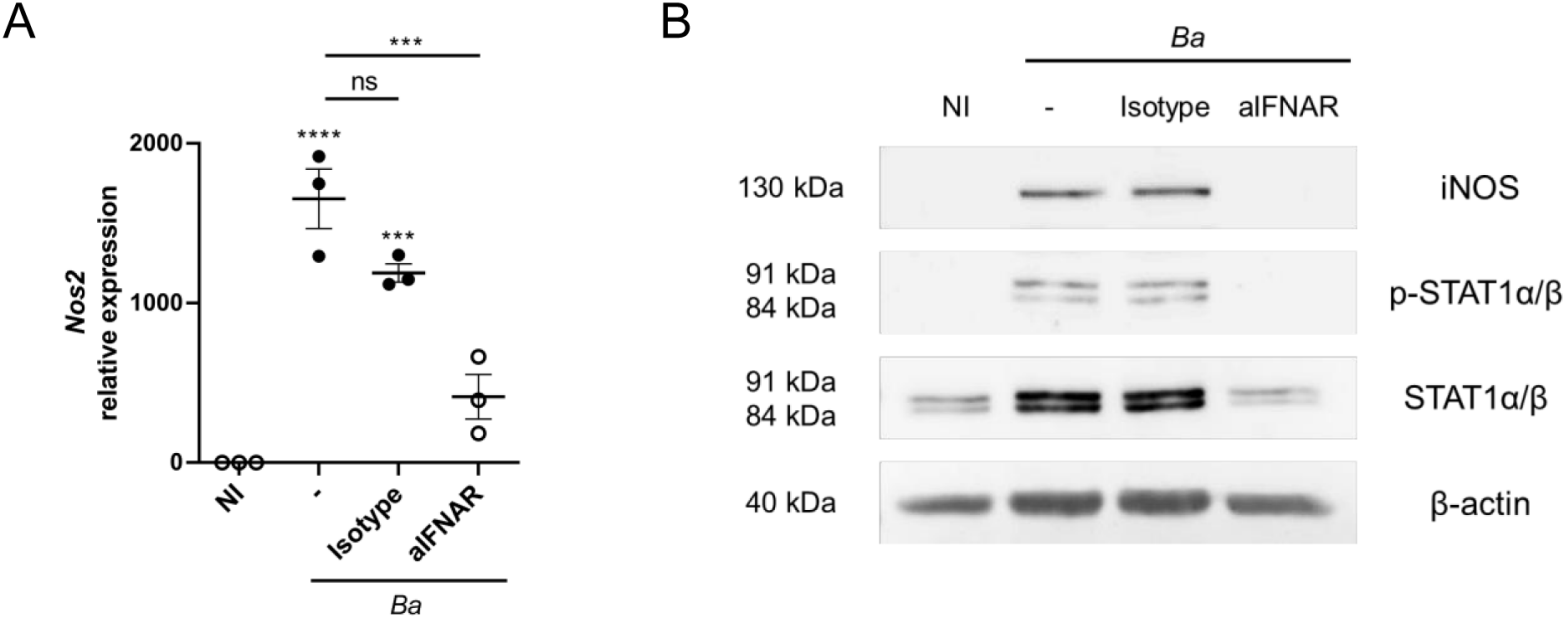
IFNAR neutralization in *B abortus*-activated microglia suppresses iNOS expression through inhibition of STAT1 expression and phosphorylation. Microglia cultures were infected with *B abortus* (*Ba*; MOI 100) in the presence of IFNAR1 neutralizing antibody (aIFNAR; 10 μg/mL) or its isotype control (10 μg/mL) for 24 h. Gene expression of *Nos2* was analyzed by RT-qPCR. Data are shown from three independent experiments as mean ± SEM **(A)**. iNOS, total and phosphorylated STAT1α/β protein expressions were evaluated by Western blot. β-actin was used as a loading control. Data shown are from a representative experiment of three performed **(B)**. ***p < 0.0005; ****p < 0.0001 *vs*. non-infected (NI) cultures, except where indicated. Non-significant (ns).

NO is generated in phagocytic cells by a chemical reaction catalyzed by the enzyme inducible NO synthase (iNOS). Modulation of the expression of this enzyme, both at the transcriptional and the post-transcriptional level, represents the main regulatory mechanism for NO production (18). Therefore, we investigated if the inhibition in the NO production caused by the neutralization of IFNAR on microglia is due to variations in the expression of iNOS. As such, microglia cultures were infected with *B. abortus* in the presence or not of IFNAR1 neutralizing antibody or its isotype control. At 24 h post-infection, both the *Nos2* gene and protein expression levels of iNOS were evaluated by RT-qPCR and Western blot, respectively. Blocking of IFNAR in microglia cultures diminished both the mRNA (**Figure 4A**) and protein synthesis of iNOS (**Figure 4B**). Binding of type I IFN to IFNAR activates the protein tyrosine kinases Tyk2 y Jak1, which phosphorylate tyrosine residues in cytoplasmic domains of the receptor. These phosphorylated sites act as docking points for the binding of signal transducers and activators of transcription (STAT) proteins, which in turn are phosphorylated by the protein tyrosine kinases. Once phosphorylated, these transcription factors form dimers that translocate to the cell nucleus, where they regulate the expression of several genes (19). The formation of different transcription factor complexes is determined, in part by the number and isoforms of STAT proteins expressed by a particular cell type. On one side, the dimerization of phosphorylated STAT1 and STAT2 recruits to IRF9 transcription factor, composing the ISGF3 complex that promotes the expression of genes with anti-viral functions, and which contain in their promoter IFN-stimulated response elements (ISRE). On the other hand, homodimers formation of phosphorylated STAT1 translocate to the nucleus and induce the transcription of genes that contain a gamma-activated sequence (GAS) in its promoter (20). Since the iNOS gene promoter contains a GAS sequence (21), we decided to evaluate the tyrosine phosphorylation of STAT1 in *B. abortus*-activated microglia by Western blot. *B. abortus* infection induces STAT1 phosphorylation in microglia, which is inhibited by blocking IFNAR (**Figure 4B**). Microglia infection not only caused the phosphorylation of STAT1, but also increased protein expression of STAT1 (**Figure 4B**), and IFNAR neutralization decreased STAT1 expression to similar levels of those observed in non-infected microglia.

### NO production in *B. abortus*-activated microglia requires the combined action of type I IFN, NF-κB and the MAPK p38 and ERK1/2 signaling pathways

**Figure 5:**
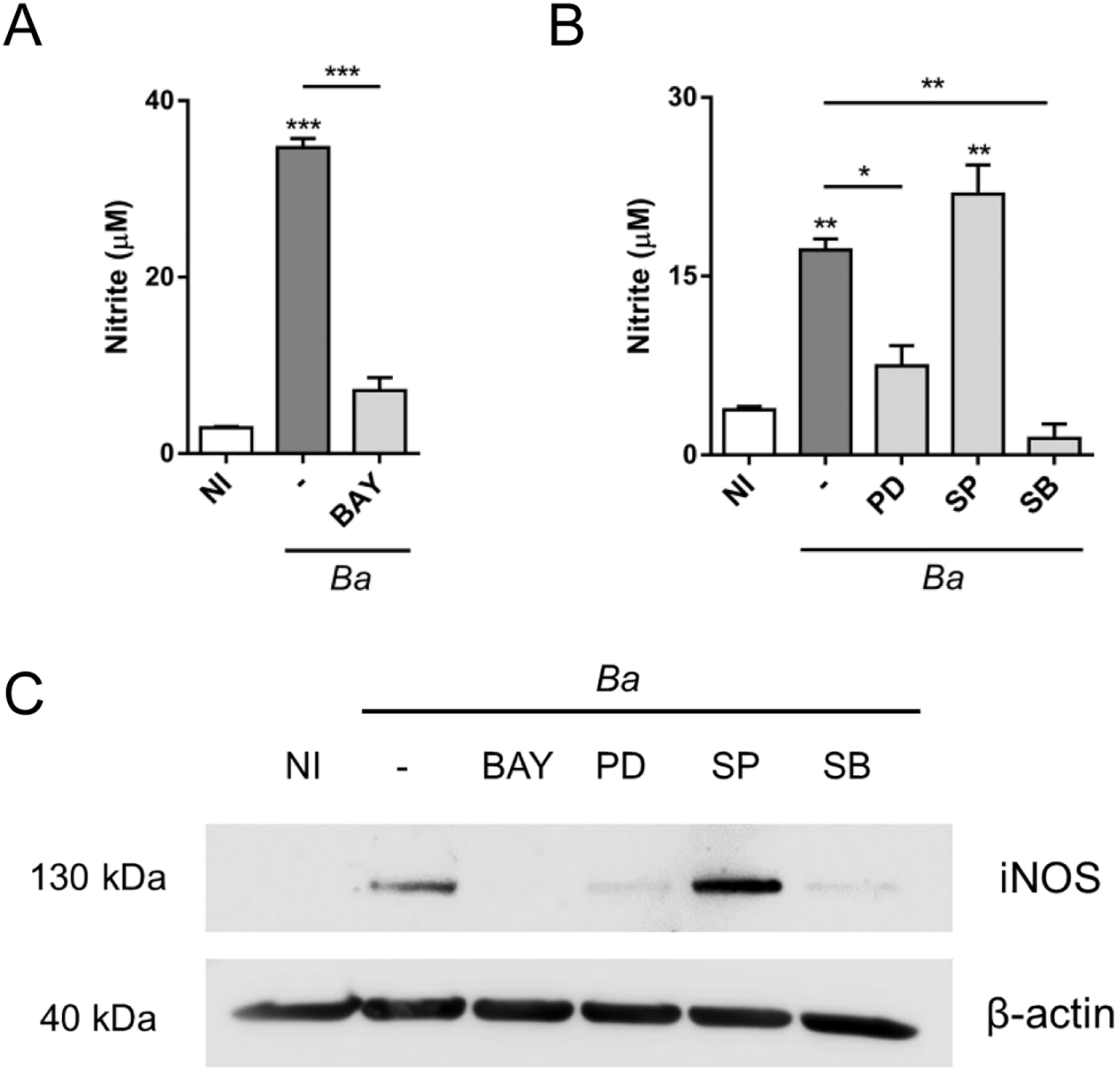
Inhibition of NF-κB, ERK1/2 and p38 affects NO production in *B abortus*-activated microglia. Microglia cultures were pre-incubated during 1 h with NF-κB inhibitor, BAY 11-7082 (BAY; 20 μM) **(A)** or with inhibitors for each one of the MAPK signaling pathways: PD98059 (PD; 50 μM; ERK1/2 inhibitor), SP600125 (SP; 10 μM; JNK1/2 inhibitor) or SB203580 (SB; 10 μM; p38 inhibitor) **(B)** and then infected with *B abortus* (*Ba*; MOI 100) for 48 h **(A, B)** or 24 h **(C)** in the presence of each inhibitor. NO production was evaluated in culture supernatants by Griess reaction. Data are shown as mean ± SEM from a representative experiment of three performed **(A, B)**. iNOS expression was evaluated by Western blot. β-actin was used as a loading control. Data shown are from a representative experiment of three performed **(C)**. *p < 0.05; **p < 0.005; ***p < 0.0005 *vs*. non-infected cultures (NI), except where indicated.

Our results show that NO production in *B. abortus*-activated microglia is regulated by type I IFN that promotes iNOS gene transcription. Reportedly, studies of signaling pathways and transcription factors involved in iNOS induction revealed a marked heterogeneity depending on the cellular type and species considered (22). It has been shown that iNOS gene promoter presents binding sites for diverse transcription factors, including sites for STAT1 binding, as already mentioned above. Among these transcription factors is NF-κB, which presents multiple binding sites in the iNOS promoter region. Several studies have shown that blocking the activation of NF-κB in macrophages results in the inhibition of iNOS expression (23, 24), indicating a key role of this transcription factor. On the other hand, studies carried out in different cell types have also demonstrated the participation of MAPK signaling pathways, although, cell dependent, with different results about which of them (ERK1/2, JNK1/2 or p38) are involved in iNOS induction (25–27). Therefore, to determine the involvement of NF-κB and the MAPK signaling pathways in the expression of iNOS in *B. abortus*-activated microglia, we used specific inhibitors and evaluated NO production by Griess reaction and iNOS expression by Western blot, respectively. The inhibition of NF-κB (using BAY 11-7082) (**Figure 5A**) or p38 (using SB203580) (**Figure 5B**) completely abrogated NO production in *B. abortus*-activated microglia. Inhibition of ERK1/2 (using PD98059) had a partial effect on NO production, whereas inhibition of JNK1/2 (using SP600125) did not affect its production (**Figure 5B**); which correlate with the inhibition of iNOS protein expression by Western blot (**Figure 5C**). These results, together with the ones obtained in **Figure 4**, indicate that type I IFN signaling, NF-κB, p38 and ERK1/2 are necessary to induce NO production in *B. abortus*-activated microglia, since selective inhibition of either of them is sufficient to affect NO production.

**Figure 6:**
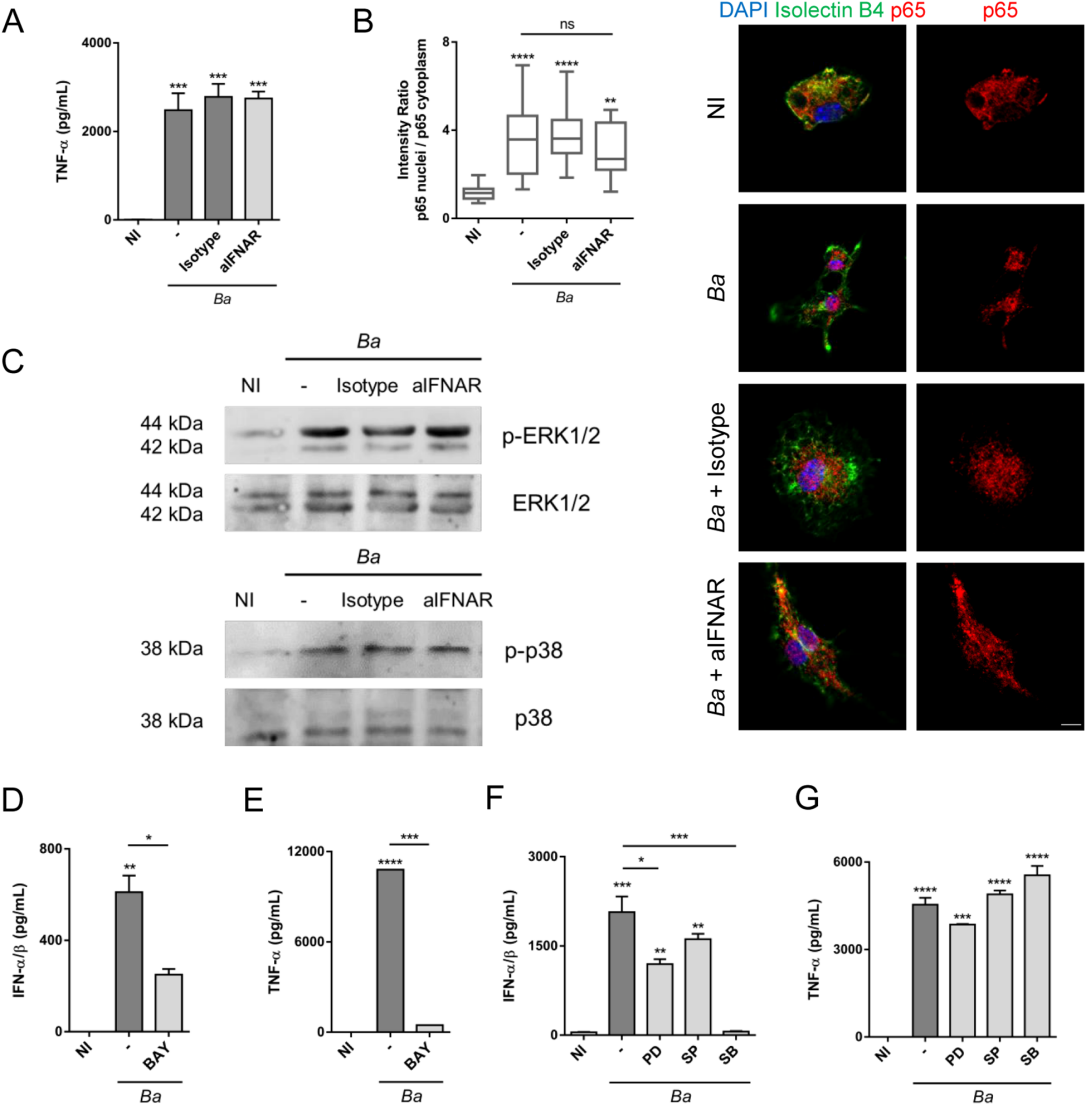
Type I IFN signaling do not modulate NF-κB, ERK1/2 nor p38 activation, but NF-κB, ERK1/2 and p38 inhibition decrease type I IFN production in *B abortus*-activated microglia. Microglia cultures were infected with *B abortus* (*Ba*; MOI 100) in presence of IFNAR1 neutralizing antibody (aIFNAR; 10 μg/mL) or its isotype control (10 μg/mL) for 24 h TNF-α secretion was measured in culture supernatants by ELISA **(A)**. Cells were fixed and labeled with anti-p65 monoclonal antibody (Red). Microglial cells were labeled with B4-isolectin (Green). For nuclear staining DAPI was used (Blue). p65 nuclear translocation was determined by confocal microscopy and analyzed by quantification of the ratio of mean fluorescence intensity for p65 staining between nucleus and cytoplasm regions for cells in each condition. Representative images are shown. Scale bar: 5 μm **(B)**. Total and phosphorylated ERK1/2 and p38 were evaluated by Western blot **(C)**. Microglia cultures were pre-incubated for 1 h with BAY 11-7082 (BAY; 20 μM; NF-κB inhibitor), PD98059 (PD; 50 μM; ERK1/2 inhibitor), SP600125 (SP; 10 μM; JNK1/2 inhibitor) or SB203580 (SB; 10 μM; p38 inhibitor) and then infected with *B abortus* (*Ba*; MOI 100) for 48 h in the presence of the inhibitor. Type I IFN production was measured in culture supernatants using the reporter cells B16-Blue™ IFN-α/β **(D, F)**, and TNF-α secretion by ELISA **(E, G)**. Data are shown as mean ± SEM from a representative experiment of three performed. *p < 0.05; **p < 0.005; ***p < 0.0005; ****p < 0.0001 *vs*. non-infected (NI), except where indicated. Non-significant (ns).

**Figure 7:**
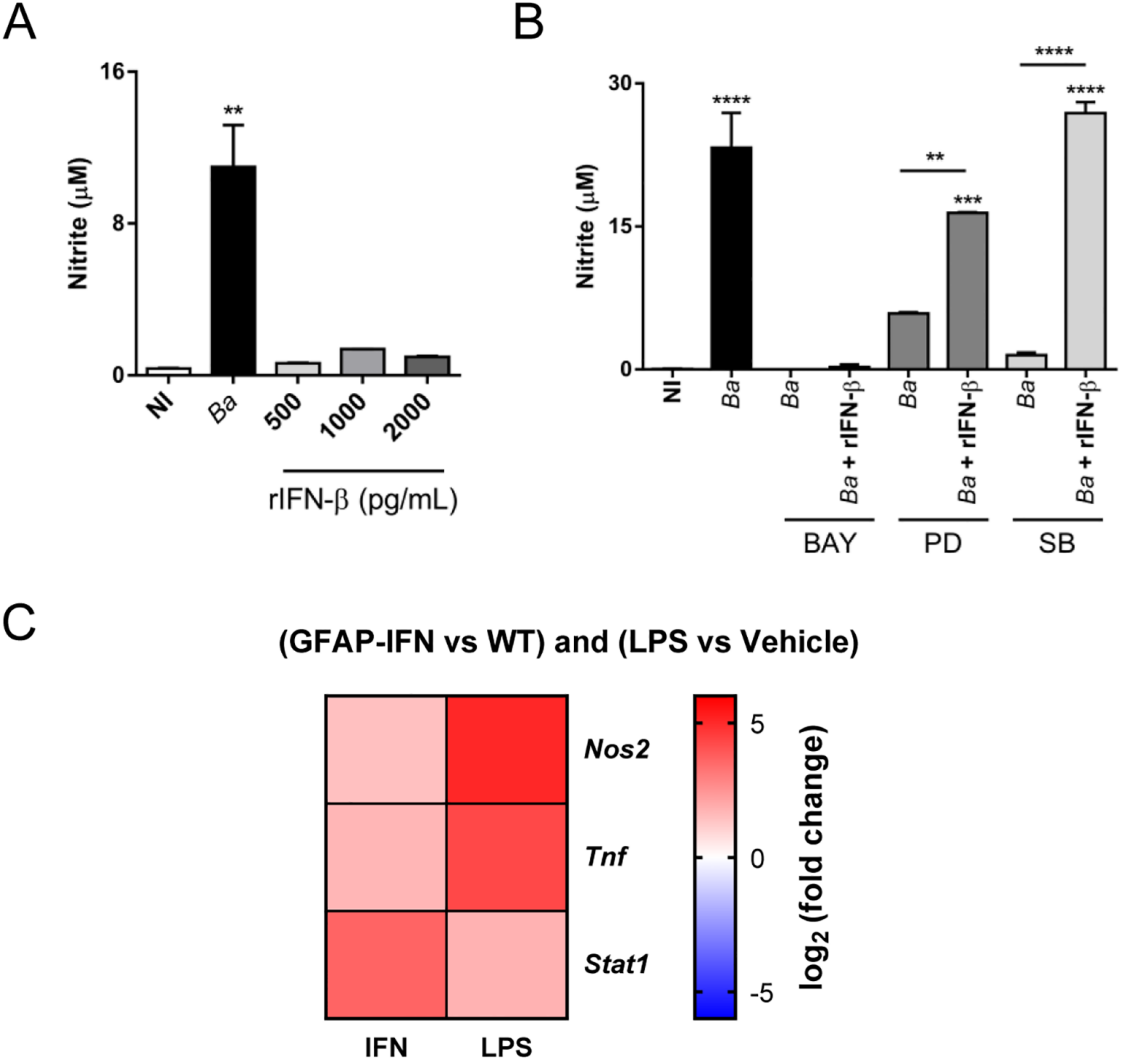
NO production by *B abortus*-activated microglia requires type I IFN signaling and NF-κB activation. Microglia cultures were infected with *B abortus* (*Ba*; MOI 100) or increasing concentrations of recombinant IFN-β (rIFN-β) for 48 h, and the NO production was evaluated in culture supernatants by Griess reaction **(A)**. Microglia cultures were pre-incubated for 1 h with BAY 11-7082 (BAY; 20 μM; NF-κB inhibitor), PD98059 (PD; 50 μM; ERK1/2 inhibitor) or SB203580 (SB; 10 μM; p38 inhibitor) and then infected with *B abortus* (*Ba*; MOI 100) in the presence of the inhibitor plus the addition or not of recombinant IFN-β (rIFN-β; 500 pg/mL) for 48 h. NO was determined in culture supernatants by Griess reaction **(B)**. Heatmap from public transcriptomic data showing the differential expression of *Nos2*, *Tnf* and *Stat1* genes in microglia from GFAP-IFN transgenic mice (PRJEB46781) and systemically LPS injected mice (GSE75246). Log2 fold change in gene expression are shown for GFAP-IFN transgenic mice and LPS injected mice compared with WT mice and vehicle injected mice, respectively **(C)**. Data are shown as mean ± SEM from a representative experiment of three performed. **p < 0.005; ***p < 0.0005; ****p < 0.0001 *vs*. non-infected (NI) cultures, except where indicated.

**Figure 8:**
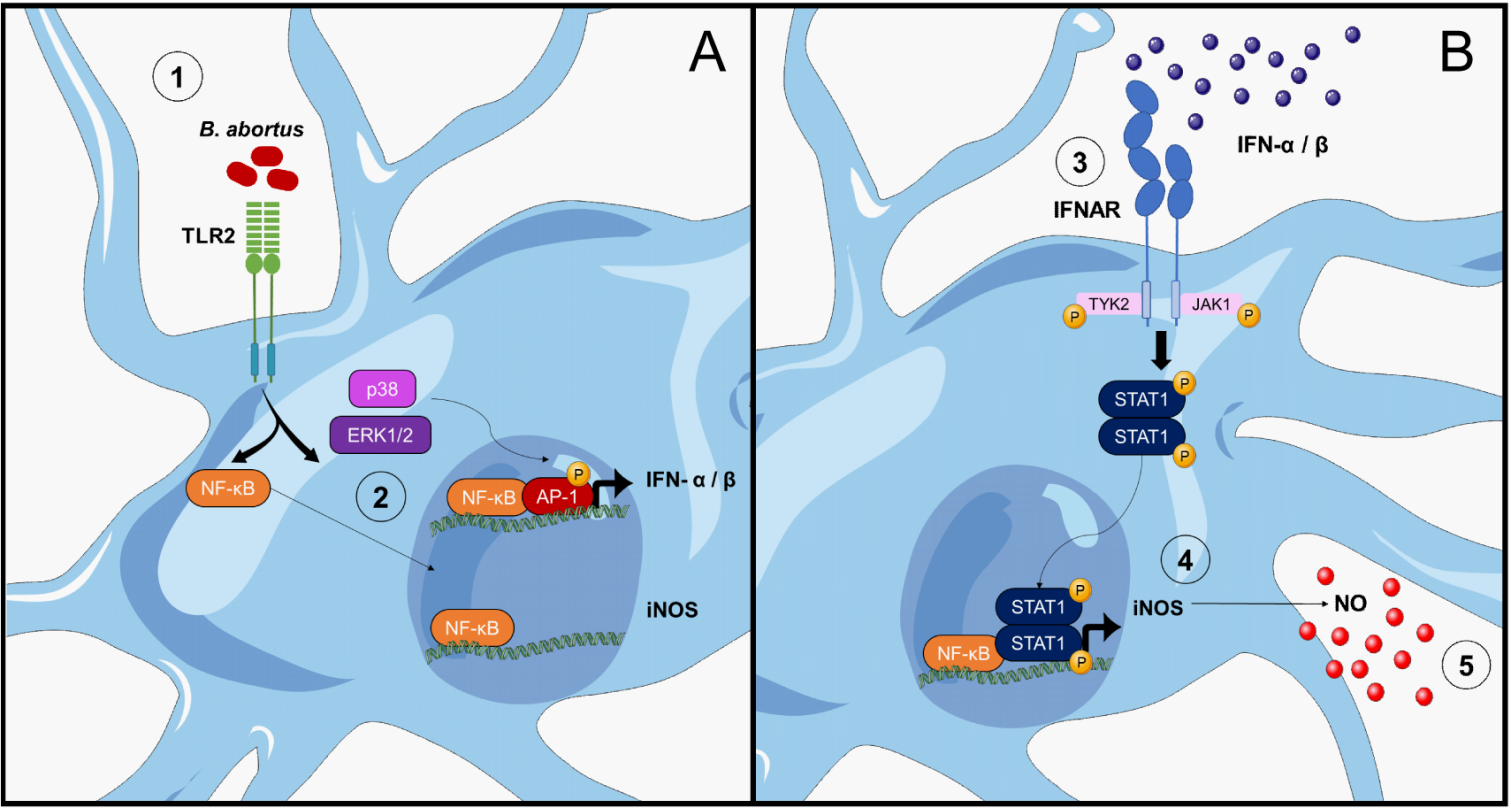
Modulation of *B abortus*-induced NO production in microglia by type I IFN. **(A)** L-Omp19 of *B abortus* is recognized by TLR2 (1), activating, on one hand, NF-κB transcription factor and on the other p38 and ERK1/2 MAPK that would lead to the activation of AP-1 transcription factor (48). Both activated factors translocate to the nucleus where they bind to IFN-α and IFN-β gene promoters, inducing their transcription. On the other hand, NF-κB also binds to the iNOS gene promoter, but is not enough to activate its transcription (2). **(B)** Once synthetized and secreted to the extracellular space, type I IFN bind to IFNAR and would activate Tyk2 and Jak1 tyrosine kinases (19), which phosphorylate STAT1 (3). Phosphorylated STAT1 form dimers and translocate to the nucleus where it binds to GAS sequences in the iNOS gene promoter (21). The combine action of NF-κB and STAT1 in the iNOS promoter activates the transcription of this gene (4). Expression of the iNOS enzyme produces NO, which is liberated by the microglia (5). Figure was created using images from Servier Medical Art, licensed under Creative Commons Attribution CC BY 4.0.

Next, we sought to investigate how these factors participate in the induction of iNOS. First, we evaluated if neutralization of type I IFN signaling affects NF-κB or ERK1/2 and p38 activation in *B. abortus*-activated microglia. For this, microglia cultures were infected with *B. abortus* in the presence or not of IFNAR1 neutralizing antibody or its isotype control, and the activation of these pathways was evaluated. We measured TNF-α in culture supernatants as an indicator of NF-κB activation, since the expression of this cytokine depends exclusively on its activation (28). Neutralization of type I IFN signaling did not modify TNF-α secretion, indicating that NF-κB activation is not affected by this pathway (**Figure 6A**). To confirm that IFNAR blocking did not influence NF-κB activation, we evaluated the nuclear translocation of p65 subunit of NF-κB. Corroborating the previous result, inhibition of type I IFN signaling did not affect nuclear translocation of p65 (**Figure 6B**). On the other hand, we investigated the activation of ERK1/2 and p38 evaluating their phosphorylation by Western blot. Activation of microglia with *Brucella* induced ERK1/2 and p38 phosphorylation, which was not affected by IFNAR neutralization (**Figure 6C**). Hence, type I IFN did not influence NF-κB nor ERK1/2 and p38 activation in *B. abortus*-activated microglia, indicating that in the production of NO, type I IFN act independently or subsequently to NF-κB and MAPK activation. Thus, we next evaluated if blocking NF-κB, ERK1/2 or p38 activation in *B. abortus*-activated microglia affects type I IFN production using the reporter cell line B16-Blue™ IFN-α/β. Inhibition of NF-κB activation with BAY 11–7082 significantly (*p* < 0.05) decreased type I IFN secretion in *B. abortus*-activated microglia (**Figure 6D**), although it did not abrogate its production. As expected, the presence of BAY 11–7082 blocked TNF-α secretion as induced by the bacterium (**Figure 6E**). On the other hand, ERK1/2 inhibition also caused a partial and significant (*p* < 0.05) decreased in type I IFN levels, whereas p38 inhibition completely blocked type I IFN secretion (*p* < 0.0005) (**Figure 6F**). As expected, JNK inhibition did not affect type I IFN secretion (**Figure 6F**). The inhibition of these MAPK did not modify TNF-α secretion (**Figure 6G**), indicating that they are not involved in NF-κB activation. Considering these results, we decided to evaluate if stimulation of microglia with type I IFN alone is sufficient to induce NO production. For this, microglia cultures were treated with increasing concentrations of recombinant mouse IFN-β - starting at a concentration similar to the one secreted by *B. abortus*-infected microglia (**Figure 1**) - and NO production was measured by the Griess reaction. We observed that none of the concentrations of IFN-β tested was sufficient by themselves to induce NO production (**Figure 7A**). Moreover, restitution of IFN-β to *B. abortus*-activated microglia in the presence of BAY 11–7082 failed to induce NO production, indicating that NF-κB activation is essential for iNOS expression independently of its effect in type I IFN production (**Figure 7B**). In contrast, the addition of recombinant IFN-β to *B. abortus*-activated microglia in the presence of ERK1/2 or p38 inhibitors restores NO production induced by the bacterium (**Figure 7B**). This result indicates that inhibition of NO production by ERK1/2 or p38 blocking occurs because there is a decrease in type I IFN production, although NF-κB is still activated in *B. abortus*-activated microglia. Overall, our findings suggest a combined action of transcription factors NF-κB and STAT1, which bind to specific sites in the iNOS gene promoter to activate its transcription. Transcriptomic analysis of available RNA-seq datasets supports our results. We analyzed microglial gene expression in available transcriptomic datasets from a chronic mouse model of IFN-α overexpression in astrocytes (13) and from a neuroinflammatory mouse model induced by intraperitoneal injection of LPS (14), and evaluated the expression of *Nos2*, *Tnf* and *Stat1* genes. In agreement with our results, fully transcriptional activation of *Nos2* (the gene that encodes the iNOS enzyme) required both NF-κB activation (reflected as an increase in *Tnf* expression) and type I IFN signaling (shown by the increase in *Stat1* expression) as occurs after stimulation with LPS, a TLR4 agonist that is widely demonstrated that induce activation of both pathways (16, 29) (**Figure 7C**). On the contrary, overexpression of IFN-α induced a significant increase in STAT1 expression, but a weak *Nos2* gene expression as this model only presents a modest increase in *Tnf* expression, indicating that it lacks a strong activator of NF-κB transcription factor (**Figure 7C**). Besides, our results indicate a cooperative action of NF-κB, ERK1/2 and p38 on type I IFN production in *B. abortus*-activated microglia as depicted in **Figure 8**.

## Discussion

Neuronal death by primary phagocytosis implies not only an increase in the phagocytic capacity of microglia, but also an increase in NO production (7, 8, 17). How this refined mechanism is regulated is not completely understood. Our previous work demonstrated that IL-6 is involved in regulating microglial phagocytic activity (8). Results presented in this manuscript demonstrate that type I IFN is involved in regulating NO production in *B. abortus*-activated microglia. IFNAR neutralization was sufficient to completely inhibit neuronal death induced by *B. abortus*-infected microglia or microglia activated in a paracrine way by infected astrocytes. A recent study has identified a population of microglia that responds to type I IFN in the developing somatosensory cortex of mice, which is involved in the phagocytosis of neurons (30). Interestingly, this work demonstrates that mice deficient in IFNAR present an accumulation of cortical neurons with breaks in double-stranded DNA, an indicator of cellular stress or damage, while they do not show changes in the number of TUNEL^+^ neurons, a marker of terminal DNA damage that leads to apoptosis (30), suggesting that this type I IFN-responding microglia are phagocytosing live (but stressed) neuron. Several congenital and acquired diseases with chronic production of type I IFN, collectively named type I interferonopathies, present neurological symptoms (31, 32). Also, type I IFN have been involved in the pathogenesis of neurodegenerative diseases as Alzheimer’s disease (33, 34) and prion disease (35). *In vitro* studies have demonstrated that microglia have a more pronounced response to type I IFN in comparison to astrocytes and neurons, indicating that microglia are the main cell type responding to type I IFN in the CNS (36, 37). Of note, it has been identified by single cell RNA-seq an increase in the population of microglia that responds to type I IFN in animal models of viral infections, cerebral tumors, Alzheimer’s disease, transient ischemia and demyelinating lesions, suggesting that type I IFN responses are present in several neuropathological states (30). Clinical evidences, in lupus patients, with elevated type I IFN in serum (38), and in patients that receive treatment for chronic hepatitis C infections and cancer (39, 40), show that peripheral IFN-α produce CNS alterations. Currently, the molecular mechanisms triggered by type I IFN and that drive CNS manifestations are motive of extensive research.

Our results demonstrate that IFNAR neutralization completely suppress NO production in *B. abortus*-activated microglia, assigning to type I IFN a key role in the production of this mediator. However, IFNAR neutralization did not affect phagocytic activity of microglia. This goes along with a report in an *in vivo* murine model of Alzheimer’s disease instead where IFNAR1 neutralization decreased neuronal synaptic loss, but did not modify the number of β-amyloid plaques accumulated in the brain (41). Moreover, IFNAR neutralization did not change the number of β-amyloid fibers phagocytosed by microglia or the expression of the lysosomal marker CD68 (used as a phagocytic marker) (41). Our results also indicate that type I IFN signaling neutralization did not affect IL-6 secretion and the phagocytic capacity of microglia; confirming that phagocytosis and NO production are independently regulated in *B. abortus*-activated microglia.

NO production in cells is tightly regulated. In comparison to oxygen reactive species that are generated very fast through the assembly of pre-formed subunits of the NADPH oxidase enzymatic complex, NO production is more slowly since cells in basal states do not express iNOS (22). In fact, previous results from our group regarding NO production kinetics in *B. abortus*-infected microglia show a significant increase in NO secretion not before 24 h post-infection (7). Regulation of NO production is very important since it was shown in diverse tissues that dysfunctional production of nitrogen reactive species might contribute to different physiopathological effects caused by damage of proteins, lipids and nucleic acids by nitration (42). Diverse studies have shown that the pathways inducing iNOS expression might vary among different cells or species. Activation of NF-κB and STAT1 transcription factors, and its concerted binding on the iNOS promoter seems to be an essential step for iNOS induction in the majority of cells (22). Results obtained here indicate that IFNAR neutralization decrease iNOS gene transcription, and therefore protein expression of iNOS in activated microglia. As already mentioned, IFNAR is a receptor associated to protein tyrosine kinases. Interestingly, it has been described inhibition of NO production induced by LPS from *E. coli* in microglia treated with tyrosine kinases inhibitors, although the identity of such kinases was not investigated (43). Considering our results, we can speculate that NO inhibition observed after treatment with these inhibitors possibly occurs by inhibition of the Jak1 and Tyk2 tyrosine kinases associated to IFNAR, which thereby suppress the phosphorylation and activation of STAT1. *B. abortus*-activated microglia increase both expression and phosphorylation of STAT1, which is inhibited by neutralization of IFNAR. This correlates with previous observations which shown that *B. abortus* induce STAT1 phosphorylation dependent on type I IFN signaling in bone marrow-derived macrophages (44). Our results indicate that STAT1 phosphorylation induced by IFNAR activation in microglia is a key event in iNOS expression, and therefore in the subsequent NO production, which is consistent with results reported by others (45, 46). It has been described that STAT1 has a central role in iNOS gene induction by direct binding to its promoter in GAS sequences, or indirectly by inducing IRF-1 expression which also possess binding sites in the iNOS promoter (22). In fact, mixed glial cell cultures from mice deficient in IRF-1 show a partial decrease in iNOS gene transcription and NO production induced by LPS (47). This same study indicates that NF-κB inhibition reduce iNOS mRNA levels as well NO production to basal levels (47). This observation agrees with our results, which also assigned a critical role for NF-κB transcription factor on iNOS expression in glial cells.

TLR activation with different pathogen-associated molecular patterns not only engages the NF-κB canonic signaling pathway, but also MAPK signaling pathways (48). Both signaling pathways act in a concerted way to induce gene transcription of cytokines needed to promote an immune response. Previous results from our group demonstrated that *B. abortus* is able to activate ERK1/2 and p38 in astrocytes (49). Results presented here indicate that activation of microglia with *B. abortus* induce ERK1/2 and p38 phosphorylation, adding new evidence of MAPK activation induced by *Brucella* in another cell type of the CNS. Although MAPK have been involved in NO production in various cell types (25–27), their mechanistic action has not been fully elucidated; with different outcomes being cell-type dependent. Our results describe a comprehensive role of the MAPK in iNOS regulation in *B. abortus*-activated microglia. ERK1/2 and p38 MAPK inhibition of *B. abortus*-activated microglia significantly decreases type I IFN secretion. Moreover, the exogenous addition of recombinant IFN-β to culture medium restores NO production inhibited by both PD98059 and SB203580 MAPK inhibitors, confirming that the role of ERK1/2 and p38 in NO production is to enhance type I IFN production in microglia. Our results demonstrate that p38 has a key role in type I IFN in *B. abortus*-activated microglia. This effect was also observed in human blood monocyte-derived macrophages, where p38 inhibition, but not JNK, decreases IFN-β expression in response to *Listeria monocytogenes* infection (50). Different authors have reported a complex role of MAPK in type I IFN regulation in macrophages, with both positive and negative effects depending on the context. In particular, it has been demonstrated that p38 through MSK1 and MSK2 kinases caused inhibition of IFN-β gene transcription, whereas via MK2 and TTP promote mRNA stability and/or translation (51). We did not observe differences in the production of TNF-α by *B. abortus-*activated microglia in the presence or absence of MAPK inhibitors, at least when added individually, and in this regard agreeing with results observed elsewhere in glial cells (52). This indicates that NF-κB and MAPK activation in microglia occurs independently, in contrast to the TNF-α decrease observed in *B. abortus*-activated astrocytes after treatment with MAPK inhibitors (49). Altogether, our results are in agreement with the notion that type I IFN production is tightly regulated and only takes place after the binding of various transcription factors to specific regulatory regions on its gene promoter. The *ifnb* promoter has four positive regulatory domains with binding sites to NF-κB, IRF3, IRF7 and AP-1 complex that comprises activating transcription factor 2 and c-Jun (53). The importance of each transcription factor in IFN-β induction depends on the context and cell type. For example, in murine embryonic fibroblasts the activation rate of NF-κB is limiting for *ifnb* gene transcription only when IRF3 activation is low (54).

Finally, our results report for the first time the ability of L-Omp19 to induce type I IFN secretion, providing new evidence of a TLR2 ligand of *Brucella* that is involved in type I IFN induction. Recent studies have shown that TLR2, similarly to what has been described for TLR4, may be internalized and transported to endolysosomal compartments where it activates IRF7 through adaptor proteins MyD88 and TRAM, inducing type I IFN expression (55, 56).

The diversity of reported signaling pathways and transcription factors involved in iNOS induction is related not only to differences in cell type and species used for investigation, but also because many of these studies were conducted using single pathogen-associated molecular patterns (usually LPS) with the addition or not of a single cytokine (IFN α/β or IFNγ), or its neutralizing antibody (45, 52, 57, 58). Our work adds a more comprehensive view of the way in which the production of NO is regulated in the course of an infectious event, describing a complex mechanism whereby a cooperative action of NF-κB, ERK1/2 and p38 must take place for type I IFN production in *B. abortus*-activated microglia. As a result of this production IFNAR signaling induces the activation of STAT-1 which acts in concert with NF-κB to induce iNOS expression and NO production (**Figure 8**), leading to neuronal death by activated microglia. Given the potential of MAPK inhibitors in anti-inflammatory cerebral treatments (59), our findings suggest that blocking these molecules could limit type I IFN secretion, offering a possible pharmaceutical approach to mitigate neurobrucellosis-related complications.

## Limitations of the study

This paper has the following general limitations: the experiments were done with mouse cells, so we do not know if the results will extrapolate to humans and human disease. The results were obtained in *in vitro* primary co-cultures of neurons and microglia and we do not know if they are extrapolable to *in vivo* situations.

## Data Availability

The raw data supporting the conclusions of this article will be made available by the authors, without undue reservation.
